# Community Barriers, Enablers, and Normative Embedding of Second Year of Life Vaccination in Ghana: A Qualitative Study

**DOI:** 10.9745/GHSP-D-22-00496

**Published:** 2023-06-21

**Authors:** Brent Wolff, Raymond A. Aborigo, Maxwell Dalaba, Joseph K. L. Opare, Laura Conklin, George Bonsu, Kwame Amponsa-Achiano

**Affiliations:** a Global Immunization Division, U.S. Centers for Disease Control and Prevention, Atlanta, GA, USA.; b Navrongo Health Research Centre, Navrongo, Ghana.; c Institute of Health Research, University of Health and Allied Sciences, Ho, Ghana.; d African Field Epidemiology Network, Kampala, Uganda.; e Ghana Health Service, Accra, Ghana.

## Abstract

**Introduction:**

Coverage rates for second year of life (2YL) vaccination still lag behind infant vaccination in most settings. We conducted a qualitative baseline study of community barriers and enablers to acceptance of 2YL vaccines in Ghana 4 years after introducing the second dose of the measles-containing vaccine.

**Methods:**

We conducted 26 focus group discussions in 2016 with men and women caregivers from mixed urban, peri-urban, and rural areas, as well as pastoralists, using semistructured topic guides based on the Health Belief Model theory. We conducted a thematic analysis of the discussion using NVivo software. We use Normalization Process Theory to contextualize results as a snapshot of a dynamic process of community adaptation to change to a well-established routine immunization schedule following 2YL introduction.

**Results:**

Routine immunization for infants enjoys resilient demand, grounded in strong community norms despite surprisingly low levels of vaccine literacy. Despite best practices like integration with the established 18-month “weighing visit,” demand for 2YL vaccination is still conditional on individual awareness and competition for limited maternal time, household resources, and other health concerns. An embedded norm that children should be fully vaccinated by 12 months originally sustained Expanded Programme for Immunization goals but now discouraged some caregivers from seeking vaccines for children perceived to be “too old” to vaccinate. Caregivers cited greater costs and inconvenience of taking older, heavier children in for vaccination and anticipated criticism from both community members and health care providers for coming “too late.”

**Conclusion:**

Closing the 2YL vaccination coverage gap will ultimately require modifying embedded norms among caregivers and health care providers alike. Time is necessary but not sufficient to reach this goal. Progress can be accelerated by increasing the level of community and institutional engagement and adapting services where possible to minimize added costs to caregivers of vaccinating older children.

## Abstract (precis)

Community perceptions of barriers and enablers to 2YL vaccination provide a snapshot of an active diffusion process as new practices gradually displace older embedded ones and practical costs and benefits of later vaccination are reevaluated.

## Teaser

**Key Findings:**

- We document community perspectives on barriers and enablers to second year of life (2YL) vaccination in Ghana in settings with high uptake of infant vaccination but still lagging coverage rates of the second dose of measles-containing vaccine 4 years after its introduction.
- Key enablers included 2YL integration into an established well-child visit and robust vaccine acceptance built on decades of observed safety and efficacy of infant vaccination programs. Key barriers included questions about whether older children benefited as much from vaccination, increased cost, inconvenience, and potential competition for time, household resources, and maternal health when 2YL visits overlapped with follow-on pregnancies.
- Some caregivers experienced veiled or direct criticism from community members and even health care personnel for bringing in children “too old” to be vaccinated, suggesting the persistence of previous norms encouraging all children to be fully vaccinated before their first birthday.

**Key Implications:**

- Building robust community demand for 2YL vaccination to the level achieved for Expanded Programme for Immunization infant vaccines requires time and active promotion to diffuse new practices and supporting norms among caregivers and care providers alike.
- Integrating a childhood vaccination option into antenatal care services could reduce competition for time and resources between maternal and child health and lower the cost of access to 2YL services.

## INTRODUCTION

One of the key recommendations from the 2011 Global Vaccine Action Plan^1^ that was carried over to the World Health Organization’s (WHO’s) Immunization Agenda 2030^2^ has been to extend the benefits of immunization to all ages through a “life course approach.” For childhood vaccination, extending the routine vaccination period beyond infancy and into the second year of life (2YL) is expected to support global vaccination targets in several ways.^3^ First, a strong 2YL platform is necessary to reach high coverage for the new, underutilized, and future vaccines. Currently, second dose of the measles-containing vaccine (MCV2); fourth dose (booster) for diphtheria, pertussis, and tetanus vaccine (DPT4); pneumococcal conjugate vaccine (PCV; meningitis A and B (MenA and MenB); Japanese encephalitis; and RTS,S malaria vaccine are all recommended after a child’s first birthday. By 2020, 179 of 195 countries had recommended MCV2—over half of them during the second year of life—and 12 of the remaining 16 countries have plans to introduce it by 2022.^4^ Second, strong 2YL platforms provide an important opportunity for catch-up vaccination by extending the period when childhood vaccinations are still offered free of charge. Third, the 2YL platform offers an additional contact point between children and the health care system that lends itself to integration with other child health services, such as growth monitoring, Vitamin A supplementation, and deworming.

However, challenges to the implementation of the 2YL policies exist at every level of service delivery—from finance and human resource development to community awareness and demand.^3^ The most readily accessible indicator of these collective challenges is the gap between coverage for MCV1 and MCV2, which is typically the first vaccine to be introduced to the routine 2YL schedule. According to WHO/UNICEF estimates of national immunization coverage for 2019, this gap was 14 percentage points (85% vs. 71%) globally, reaching as high as 38% for the WHO Africa region. By 2021, it had fallen to 10 percentage points (81% for MCV1 versus 71% for MCV2) due to introductions of MCV2 in several countries, including Ethiopia. In Ghana, which boasts one of the strongest immunization systems in the region—coverage for TB with bacille Calmette-Guérin vaccine, third dose of the diphtheria-pertussis-tetanus vaccine and first dose measles vaccine (MCV1) remained above 90% through the COVID-19 pandemic and have strengthened further since 2020.^5^ MCV2 coverage improved considerably since its introduction in 2012 (52%) but still lagged MCV1 coverage by 9 points in 2019 (92% versus 83%) and 11 points in 2021 (94% versus 83%).^5^

The Ghana Second Year of Life (2YL) Project was a collaboration between the Ghana Health Service and the U.S. Centers for Disease Control and Prevention (CDC) to identify immunization system barriers and implement targeted interventions to improve the second year of life vaccination in this West African country.^6^ Before starting the project in 2016, baseline assessments in implementing regions of Ghana were conducted based on a household survey,^6^ health facility assessment, and the qualitative baseline study discussed later. Description of the broader study design and implementation have been published separately.^6^^,^^7^ The current analysis aims to capture caregiver perspectives on 2YL expansion 4 years after the introduction of MCV2 as a snapshot in time of an ongoing process of community-level normalization of innovative immunization practice.

## METHODS

### Study Sites

The study was conducted in the 3 regions of Ghana (Greater Accra, Volta, and Northern regions) selected for the planned intervention phase based on lower-than-expected MCV2 coverage due to a range of different programmatic challenges.^6^ For example, Greater Accra typifies large urban characteristics of high residential mobility, higher education levels, heightened opportunity costs for caregivers’ time, and weakened influence of extended family and local community influences, among other factors. Access barriers to immunization services for populations around Lake Volta contribute to lower immunization coverage in Volta region. Northern region is among the poorest, least educated, and most sparsely populated areas in Ghana, where the presence of seasonally mobile pastoralist groups poses particular challenges for routine immunization services.

### Sample Selection

We conducted 8 groups per region, with an additional 2 groups for pastoralists in the Northern region for a total of 26 groups (Table 1). Each group consisted of 6–15 participants, with a median of 8 and a total of more than 220 participants. Purposive sampling was conducted in 2 stages, first for community selection and second for participant selection. Anticipating that access barriers to health information and immunization services strongly determine acceptance and uptake of 2YL vaccination, we selected 1 urban and 1 rural site in each region for data collection and added a second rural site in the Northern region to include pastoralist groups. Aside from pastoralist groups who were interviewed close to their temporary encampments, all groups were recruited from preschool catchment areas convenient to the schools where discussions were held. Four focus group discussions (FGDs) were planned within each catchment area. Two of the 4 groups were devoted to mothers with children currently enrolled in local preschools as the modal category of primary caregivers responsible for taking children for immunization in such settings. Additional groups with fathers of enrolled preschool children and mothers of preschool-aged children not enrolled in preschools were included in the design to detect contrasting perspectives on immunization by gender and engagement with formal education, respectively.

**TABLE 1.** Focus Groups Conducted by Region and Other Break Characteristics, Ghana Second Year of Life Project Qualitative Baseline Study, 2016

	Region			
	Northern, No.	Volta, No.	Greater Accra, No.	Total, No.
Urban	4	4	4	12
Peri-urban^a^/Rural	4	4	4	12
Pastoralist	2	--	--	2
Fathers	3	2	2	7
Mothers	7	6	6	19
Parents of in-school children	8	6	6	20
Parents of out-of-school children	2	2	2	6
Total	10	8	8	26

a Peri-urban groups were limited to mixed urban and rural settlements outside Accra, Ghana.

Within study settings, selection of discussion participants was purposive within eligibility criteria of being a parent of preschool-aged children, children’s enrollment status, and ability to access the meeting place and participate actively without dominating the group discussion. Local teachers and health volunteers were appointed by district health officials or pastoralist leaders as mobilizers to recruit 10 participants “typical” of the school catchment area where they were recruited (e.g., capturing a usual range of residents, not necessarily the best educated or most outspoken) for each group. Mobilizers followed written scripts to describe the general study topic, time, location, and expectations for participation. At the beginning of each discussion, moderators first provided information about the study purpose and how the information would be used and then asked participants for their oral consent to have focus group discussions recorded and analyzed after removing participants’ names. No refusals to participate were recorded during the consent process.

### Ethical Approval

Both CDC and the Ghana Health Service determined that this qualitative data collection and analysis was a minimal-risk public health program activity and not human subjects research.

### Selection and Training of Data Collectors

Four field staff with graduate-level training and experience conducting qualitative research and fluency in 1 or more local languages in their study districts received 3 days of training on the study protocol, discussion guides, and standards for transcription and translation. The training was facilitated by the CDC team with support from 2 researchers from the Navrongo Health Research Centre. One pilot discussion was conducted in Northern region at the outset of fieldwork to assess and adjust the flow of questions and probes. Because no changes were introduced to the guides based on the pilot, this focus group was counted as an urban women's focus group and was included in the final analysis.

### Topic Guide Development and Field Adjustment

Topic guides were based on 5 constructs of the Health Belief Model, designed to systematically document individual considerations that lead to vaccination decisions and actions: (1) perceived risk and susceptibility of vaccine-preventable diseases (VPDs), (2) perceived severity of disease, (3) perceived risks and benefits of vaccines, (4) perceived barriers to vaccination during the second year of life, and (5) cues to action.^8^ Additionally, participants were asked about subjective norms and other social considerations around vaccination and solicited for ideas on potential strategies to improve demand for 2YL vaccination.

### Data Collection

Two field teams conducted 26 FGDs in total. Each field team consisted of 2 field staff, 1 PhD researcher from Navrongo Health Research Centre, and 1 researcher from CDC. The Navrongo Health Research Centre researcher for each team served the role of supervisor to ensure fidelity to the study protocol, as well as quality and consistency of data collection. Field staff rotated between moderating and notetaking for discussions. Topic guides were not translated from English to give moderators flexibility in how to introduce and transition between topics while maintaining group rapport and a naturalistic flow to discussion. Daily debriefing sessions between research coordinators and field staff were conducted at the end of each day. As a result, the topic guide evolved over the first week of fieldwork when both teams were still working together to add or adjust topic questions and probe points. Each FGD lasted about 1 hour.

### Data Analysis

Field staff were responsible for typing notes from each group at the end of each day and transcribing audio-recordings into English with the assistance of discussion notes. After reviewing transcripts for clarity and completeness, supervisors imported transcripts into NVivo 10 (QSR International) for coding and thematic analysis. Starter codes were generated deductively from the FGD guide. Emergent codes were added, and a codebook was developed by supervisors after reading all the transcripts, agreeing on coding categories, then independently coding 2 interviews and comparing kappa scores, and repeating the process with the next 2 transcripts until an acceptable kappa score was attained. The first set of coding produced a kappa score of 4.5. The kappa score increased to 7.4 for the next pair of transcripts after discussion between the coders.

## RESULTS

Collection of demographic data from participants before the focus group discussion was generally limited to eligibility criteria. However, in most areas, participants were also asked questions about education level and number of children as an internal quality check to ensure participants reflected a typical range of caregivers for each community. As shown in Table 2, number of children showed little variation across settings, with both mothers and fathers reporting a median of 3 children and an interquartile range of 2 to 4 children for mothers and 2 to 5 children for fathers. Average education levels varied more widely in line with strong contrasts in access to formal education within the study setting. Male participants, those from urban areas or from Greater Accra reported higher levels of education than female participants, rural and pastoral residents, or those from Volta or Northern regions. These findings were generally in line with expectations and suggested that the mobilization strategy succeeded in recruiting participants of varied educational backgrounds typical of the surrounding settings. The results suggest a disproportionate selection of mothers with higher education levels compared to other regions, but it is not clear whether this reflected mobilizer bias to favor more “presentable” female participants in this region or a self-selection bias of educated mothers to agree to mobilization and participate in discussions.

**TABLE 2.** Focus Group Participant Characteristics, Ghana Second Year of Life Project Qualitative Baseline Study, 2016

	Female Participants					Male Participants				
	Average Education Level					Average Education Level				
	Primary School or Lower, %	Junior SS, %	Senior SS or Higher, %	Total, %	No. of Groups, (No. of Participants)	Primary School or Lower, %	Junior SS, %	Senior SS or Higher, %	Total, %	No. of Groups, (No. of Participants)
By gender	32	53	15	100	18 (148)^a^	37	33	30	100	7 (67)
By gender and region										
Accra	19	60	21	100	6 (48)	0	63	37	100	2 (19)
Volta	27	68	5	100	6 (52)	31	50	19	100	2 (16)
Northern	55	25	20	100	6 (48)^a^	63	6	31	100	3 (32)
By gender and residence										
Urban	23	56	21	100	8 (57)^a^	25	17	58	100	3 (24)
Rural	33	55	12	100	9 (82)	31	51	17	100	3 (35)
Pastoralist	89	11	0	100	1 (9)	100	0	0	100	1 (8)
**No. of children, Quartiles**	**Min./Max.**	**Median**	**1st Quartile**	**3rd Quartile**	**Groups, No. (Participants, No.)**	**Min./Max.**	**Median**	**1st Quartile**	**3rd Quartile**	**Groups, No. (Participants, No.)**
By gender	1/8	3	2	4	16 (136)^b^	1/14	3	2	5	6 (58)^b^

Abbreviations: Max, maximum; min, minimum; SS, secondary school.

a Data on education was not collected for the 1 pilot interview conducted among urban women in Northern Region.

b Data on number of children was not collected in 3 rural female groups and 1 urban male group.

### Key Enablers to 2YL Vaccination

#### Perceived Risk and Severity of VPD

Discussions about the perceived risk of VPDs in the communities naturally expanded into addressing disease severity and the value of vaccination. FGD participants gave credit to long-standing infant vaccination programs for reduced mortality and general improvements in child health.

*Childhood illness kills children very fast… We don’t even care about which type of vaccines they give to the children so far as it is one of the childhood vaccines the child gets it.* —Urban woman, Dabokpaa District, Northern Region

FGD participants gave credit to long-standing infant vaccination programs for reduced mortality and general improvements in child health.

However, in the context of 2YL vaccination, the participants of the rural FGDs perceived older children to be less vulnerable to infection and more able to withstand illness. For some, this seemed to weaken a key justification for 2YL vaccination compared to routine infant services.

*That is what my colleague said earlier, if the child is a bit grown, like after 1 year, she/he doesn’t fall sick easily compared to when the child is below 1 year.* —Rural man, Aboabo District, Northern Region

#### Perceived Benefits and Risks of Vaccines

As an extension of the strong consensus among the participants across all FGDs regarding the dangers of VPD for children, there was also consensus around the benefits of childhood vaccination itself. Some participants could point to their own experiences to vouch for the effectiveness of vaccination in terms of reduced time, money, and worry over sick children.

*When I was a child, I was vaccinated … it has made me healthier and till now, I don’t easily fall sick. For that matter, I don’t joke [about] vaccinating my children. And it has benefited me in so many ways. My children do not easily fall sick too. It means that the little money I have will not go into treating my children but for other important things. —*Pastoralist man, Gonja North District, Northern Region

### Perceived Barriers to 2YL Vaccination

Only 2 groups mentioned vaccine injury, including partial paralysis and a rumored child death after vaccination. Even when serious adverse events were mentioned, they were framed as exceptions to the general benefits of vaccines rather than a reason to refuse or delay vaccination.

Perceived barriers to 2YL vaccination can be summarized into themes of knowledge barriers (e.g., lack of familiarity with 2YL vaccines and schedules), unique access barriers to 2YL vaccination for caregivers with older children, concerns over side effects from the 2YL and catch-up vaccinations scheduled at the 2YL visit, and normative costs (e.g., real or anticipated criticism from community members and health care providers). We did not address the costs of vaccination (e.g., money, distance, means of transportation, and purchasing clothing appropriate for a visit to the clinic) that apply equally to the first- and second-year vaccination in this analysis.

#### Lack of Familiarity With Vaccines or 2YL Vaccine Schedules

Many of the educated participants from urban areas knew the names of the vaccines and the diseases they are intended to prevent, while parents in rural areas did not. Participants noted that descriptions of vaccines and diseases they prevent were included in routine vaccination cards, but these were only beneficial for those who could read. Participants complained that health care workers tended to only emphasize the dates for the next appointments and had little time or patience for providing more information about specific vaccines or answering caregivers’ questions.

*They always say, 6 weeks we should come for the injection, but we don’t really know the name of the injection. They don’t tell us the particular reasons why they are giving the baby the injection.* —Peri-urban woman, Prampram District, Greater Accra Region

Some participants complained that health care workers did not explain the new schedules to caregivers, especially for the 2YL vaccines.

*The reason is that some nurses … don’t tell us that this is the time your child is supposed to take this (2YL) injection. That is why we do not bring our children for vaccines at that age because we don’t know.* —Rural woman, Gonja North District, Northern Region

#### Unique Access Barriers for 2YL Vaccination

Bringing heavier and harder-to-manage toddlers to clinics was discussed as an extra burden of 2YL vaccination, especially in rural areas where mothers sometimes had to travel by foot. Another consideration mentioned by men and women was potentially competing demands between added vaccination visits in the second year and the arrival of the next pregnancy.

*Sometimes how to carry the children to the facility is a problem. . . . When the child is more than 1 year the mother would be pregnant and so she would get tired carrying the child and the pregnancy. So they give up on vaccinating the child to concentrate on the pregnancy.* —Pastoralist man, Gonja North District, Northern Region

Rural groups emphasized the time and effort needed to travel long distances, sometimes on foot, as important barriers for 2YL visits. Similarly, both urban and rural participants mentioned the frustration and anger of being turned back because vaccines were out of stock or health care workers would not open a new vaccine vial until more children were present. While such concerns were also cited as barriers to infant immunization, the additional effort required to bring an older, heavier child seemed particularly discouraging to participants.

*They sometimes tell us that the drug is finished and other times too they will say they can’t open [the vial] so we should go and come back later. They were doing that until I got fed up…* —Peri-urban woman, Prampram District, Northern Region

#### Concerns About Catch-up Vaccinations and Side Effects

The 2YL visit was generally referred to by the participants of the FGDs as “weighing” because it integrates routine 18-month weighing services with other services, including Vitamin A supplementation, deworming, and vaccination. In cases where infant vaccination series are incomplete, catch-up vaccinations are also delivered. The prospect of multiple vaccinations at the weighing visit invoked anxiety in many parents concerning the side effects for children, lost days of work, and anticipation of feeling blamed by health care workers for missing doses.

*Some caregivers never go for weighing. When they go late, the nurses have no choice but to inject the children with all the required vaccines which cause the child to breakdown. . . . The parent is to blame for the delay.* —Urban woman, Kpando, Volta District

*When for example your child didn’t go for vaccination this month and you go for the next 1, they combine both and give [it] to the child. Hmmm, the child cries paaa (a lot) and we have sleepless nights; their temperature goes so high you just don’t know what to do.* —Rural man, Afadjato South, Volta Region

The prospect of multiple vaccinations at the weighing visit invoked anxiety in many parents concerning the side effects for children, lost days of work, and anticipation of feeling blamed by health care workers for missing doses.

Generally, the FGD participants described the side effects of any childhood vaccination as one of the expected costs of protection against feared childhood diseases. The pentavalent vaccine, which combines DPT, hepatitis B, and Hemophilus influenzae type b and is given in a 3-dose series, earned the nickname *baatoro*, meaning “loose skirts” for keeping mothers up through the night. Since the benefits of vaccination for older children were already a matter of debate, some parents questioned whether the 2YL booster was worth the anticipated aftermath.

*Some people think after 1 year those vaccines are not so necessary. Due to their side effects, some of the injections even leave a mark on the child and all these have made some caregivers not to vaccinate their children.* —Urban man, Aboabo District, Northern Region

#### Anticipated Negative Judgment From Health Care Workers and Community Members

When asked about a maximum acceptable age to vaccinate, the general response was that as long as the vaccination was effective, there should be no age limit. However, there were substantial differences between 2YL and routine infant vaccination in terms of normative expectations of parents from health care workers and community members. Participants expressed a moral responsibility that children ought to be fully vaccinated. Caregivers who refuse to take their children for vaccination were reported in FGDs to be labeled as “wicked,” “lazy,” or “unreasonable.”

*Because of vaccines, we don’t see all these diseases in our community anymore. Now choose between health and illness and you go for illness, that makes you unreasonable!* —Pastoralist man, Gonja South, Northern Region

*They will think that you are not responsible, and you are a lazy woman who doesn’t care about your child’s health*. —Pastoralist woman, Gonja South, Northern Region

However, in the context of 2YL vaccination, a recurring theme across all FGD participants was that children older than age 12 months were “too old” to be vaccinated. Bringing in older children for vaccination evoked fear of negative judgments from others in the community and clinic waiting room.

*As for me, those children are too old to vaccinate and too heavy to carry so I don’t see the need to vaccinate such a child.* —Pastoralist woman, Gonja South, *Northern Region*

*To me, I have heard people say, “why are you carrying this old man,” for vaccination and this discourages the mothers.* —Rural woman, Afadjato District, Volta Region

Caregivers also anticipated criticism from health care workers for bringing in older children who might resist vaccination or when the mother was showing signs of her next pregnancy.

*Madam nurse, how the nurses treat them especially when they are pregnant and bring their wards for weighing, sometimes is very bad. They insult and say all kinds of things, like did we ask you to go and have sex; why?* —Peri-urban man, Prampram District, Greater Accra Region

*They will yell at you especially when you are going for the injection and you don’t position the baby well or don’t know how to position the baby.* —Peri-urban woman, Prampram District, Greater Accra Region

### Cues to Action and Recommendations for Vaccination Services

#### Improved Communication Outreach

Channels of communication for vaccine information included friends, churches and mosques, and the media—both radio and television. Chiefs and the elders were identified in the Volta region as being instrumental in the spread of information on vaccinations, especially around the time of vaccination campaigns. Participants suggested better use of traditional information channels, such as chiefs and elders, to promote 2YL vaccination.

Participants suggested better use of traditional information channels, such as chiefs and elders, to promote 2YL vaccination.

#### More Accessible Vaccination Services

Participants proposed a range of solutions to transportation challenges, particularly those facing caregivers of older children. In the short term, periodic village outreach might be needed. In the long term, expanding the number of clinics or reimbursing transportation fees on clinic days might be needed. Parents were supportive of providing vaccination services through preschools as a way to reduce the time burden on busy working mothers, provided that vaccine information could be coordinated with local clinics to avoid children receiving the same vaccines a second time at their next routine visit.

#### Improved Quality of Vaccination Services

Participants also emphasized the need for more information and more respectful treatment from providers. No child who reaches the clinic should be denied vaccination because a provider refuses to open a new vaccine vial. Health care workers should be trained to treat caregivers with more respect and patience and learn to be more responsive to their questions and concerns, regardless of education level or ethnic background. More information should be provided to caregivers about vaccines besides the date of the next visit. Information could include the name of the vaccine, the importance of the vaccine to the child, reassurance about any potential side effects, and guidance on how to manage them. Some groups suggested providing free painkillers and limiting the number of injections given at a single visit to lighten side effects. Participants thought that such efforts to improve quality of care would make mothers more confident about the vaccines and increase uptake at all visits through the routine schedule.

## DISCUSSION

In this qualitative study of barriers and enablers for 2YL vaccination in 3 underperforming regions of Ghana, we found robust acceptance of established infant immunization programs but uncertainty about the benefits and wariness of added burdens associated with vaccine visits for older children. Our findings also provide a snapshot of embedded norms governing both community demand and immunization provider practices in active transition to accommodate 2YL expansion.

From a cost-benefit perspective provided by the Health Belief Model framework, all findings point to weaker motivation to complete the 2YL vaccine visit compared to the better-established infant schedule. Regarding susceptibility and severity of VPDs, older children were perceived as more resistant to infection and less vulnerable to severe outcomes than infants. Regarding benefits of vaccination, there was broad consensus about the gains in child health since vaccination programs were introduced but less clarity around added benefits of additional vaccine doses and more individual-level calculation by parents deciding whether to attend.

Regarding costs of vaccination broadly defined, participants described what might be called a substantial price increase for the 18-month routine visit compared to infant visits. Despite the best practice of integrating 2YL vaccination visits with a scheduled well-child (“weighing”) visit, getting to and from the clinic and managing the anticipated ordeal of post-vaccination side effects were seen as more taxing with older children. Both men and women raised the additional issue of 2YL visits potentially conflicting with antenatal care demands for the next pregnancy that would place the health care needs of the mother and her older child in competition. Finally, cues to action embedded in the immediate social environment that provide regular reminders and that convey normative support for infant vaccination were less evident for 2YL vaccination visits, leaving caregivers to provide their own rationales and reminders to attend.

Stories of stock-outs or refusal to open vaccine vials and rude or indifferent treatment by health care providers elicited strong frustration that made some parents question whether it was worth returning to complete the child’s scheduled vaccinations. While not unique to the 2YL platform, these concerns were exacerbated by the extra time and trouble of bringing older children and anticipated criticism from other caregivers and health care providers for bringing children who looked “too old” to be vaccinated. Such evidence suggests that a process of transition away from embedded practices should be anticipated and actively promoted among health sector personnel in parallel with caregivers in surrounding communities.

Stories of stock-outs or refusal to open vaccine vials and the reported rude or indifferent treatment by health care providers elicited frustration from parents that made some question returning to complete the scheduled vaccinations.

In their review of Expanded Programme on Immunization (EPI) vaccine introductions, Wallace and colleagues observed that the pattern of uptake after vaccine introduction follows a threshold-saturation model,^9^ where uptake spreads slowly until reaching a critical threshold level of acceptance, followed by a period of more rapid expansion that slows again as coverage approaches a saturation level depending on regional context.^10^ A recent review of 2YL expansion suggests a similar pattern of gradual convergence over time with coverage levels of more established vaccines. For example, Peck and colleagues found that among 165 countries that had introduced MCV2 and reported an MCV2 estimate, the largest difference between MCV1 and MCV2 coverage (17%) was estimated among 34 countries that introduced MCV2 during 2010–2017 (including Ghana), compared with 5% among 131 countries that introduced the second dose before 2010.^11^ The coverage gap may be even wider for newly introduced vaccines or boosters requiring visits outside the existing vaccination schedule. A recent example that combines both conditions is the RTS,S malaria vaccine that requires 3 monthly visits starting in the fifth or sixth months and a fourth visit 12 to 18 months later.^12^ In pilot data from Ghana, Malawi, and Kenya, uptake of the fourth dose of RTS,S dropped by more than 30 percentage points compared to the first 3 doses.^13^^,^^14^

The characteristic threshold-saturation pattern is consistent with an underlying process of innovation and diffusion that has been used to describe the pattern of adoption of behavior change interventions at a population level.^10^ According to Roger’s innovation-diffusion model, the final stage of diffusion coincides with “normalization” at the social level, where new practices become “embedded into matrices of already existing, socially patterned, knowledge and practices.”^15^^,^^16^ What enhances or inhibits the embedding of new practices has been the focus of intensive theory development around translational program research and is central to the goals of 2YL expansion.^16^^–^^20^ Accordingly, we frame our interpretation of cross-sectional findings on 2YL assimilation within one of these broader explanatory models, Normalization Process Theory (NPT).

We selected NPT because it captures the socially normative aspect of childhood vaccination while providing an appropriate theoretical foundation for nesting recommendations within a broader process of population- and systems-level changes over time.^16^ In brief, NPT is a sociologically informed theory designed to understand how new material practices become embedded and ultimately integrated into the community or organizational settings.^16^ It relies on 3 central assumptions: (1) embedding requires collective work of implementing, participating, or encouraging others to do the same; (2) embedding requires time to root itself within the community or organizational settings; and (3) embedding is modeled as the product of 4 generative mechanisms for creating the conditions for the demand for new practices (coherence and cognitive participation), and the work involved in implementing changes while reconciling with existing practices (collective action and reflexive monitoring).^21^

From an NPT perspective, one might conclude that the “work” of creating conditions for the demand for 2YL vaccination in Ghana has already been accomplished through the strong foundation of trust in infant vaccination extending from the largest urban centers to the most remote pastoralist settings. The incomplete knowledge of specific vaccines and VPDs that study participants complained about was also identified as a barrier to vaccination in research conducted nearly 20 years before^22^ but has not prevented Ghana from achieving nearly universal vaccination levels in the meantime. Diffusion theories might predict that information interventions will be important at the early stages of program innovation to convince a core of early adopters. The majority of later adopters are more likely to be persuaded by the behavior of others.^23^

A remaining ideational barrier to 2YL acceptance in Ghana appears to be the persistence of a previous norm, carefully constructed over decades of past EPI programming, that equates full vaccination with infant vaccination and requires reassessment of the costs and benefits of additional visits. Building new norms and loosening the grip of the old ones will require persistent community engagement across a variety of channels, not only limited to brief radio and television announcements favoring urban audiences, to diffuse the message that complete vaccine protection is determined by the schedule, not the age of the child. As the level of social embeddedness increases, reminders and other cues-to-action begin to emerge spontaneously through horizontal networks of person-to-person interaction that create more self-sustaining demand.^20^

The greater part of the work required to narrow the 2YL coverage gap remains in the practical domain of implementation. Integration of 2YL vaccination into an already established point of contact, as Ghana has done with the 18-month weighing visit, is an effective strategy to embed any new practice. Providing such services during weekends and later hours would go further to reduce competition for time and income-earning opportunities, particularly in urban areas. Integrating vaccination services into routine antenatal care could reduce the dilemma faced by pregnant mothers between attending to their own health needs and those of their vaccine-eligible children as well as providing additional opportunities for catch-up vaccination. Implementation planning should anticipate a period of enhanced supervision to ensure vaccinators are giving supportive messages to parents regardless of the size or age of the child rather than chiding them for bringing children in “too late.” Even simple version control of promotional material and recording forms would represent important progress in settings where outdated messages from the pre-2YL era coexist with updated versions.

The greater part of the work required to close the 2YL coverage gap remains in the domain of implementation.

The active process of reflexive monitoring by which ideas are evaluated, adapted, and, ultimately, either rejected or accepted and embedded in routine practice emerges from discussions about the costs and benefits of the additional 18-month visit. As Greenhalgh and colleagues noted^20^:

*People are not passive recipients of innovations. Rather… they seek innovations, experiment with them, evaluate them, find (or fail to find) meaning in them, develop feelings (positive or negative) about them, challenge them, worry about them, complain about them, “work around” them, gain experience with them, modify them to fit particular tasks, and try to improve or redesign them, often through dialogue with other users.*

Negative interactions with health care providers cited by most participants, whether from refusal to open new vaccine vials, rude treatment from health care workers, or inattention to caregiver questions and concerns, provide an important reminder that health systems also face collective learning curves of varying steepness and duration when adopting innovative practice.^27^ Even though Ghana adopted WHO-recommended policies on safe reuse of multidose vaccine vials^24^ before 2YL expansion, vaccinators were still turning away caregivers to avoid opening new vials by the time of data collection. Participants suggested training health care workers to treat clients respectfully and avoid critical commentary that may reinforce outdated standards of practice. Need for additional training also emerged from the parallel baseline study by Nyaku et al. that found a nearly even mix of correct and incorrect knowledge about 2YL vaccination policies regarding catch-up vaccination among health care workers.^6^ A capacity-building intervention in the same 3 districts after this baseline assessment found a moderate but not statistically significant improvement in knowledge of EPI policies, data management, and communication skills, with the strongest gains in policy knowledge and the weakest gains in communication.^7^ Recent literature in quality improvement suggests a more effective strategy would be systems-level interventions to clarify job descriptions and improve supervision practices rather than relying on training alone.^25^^,^^26^ This study contributes to the relatively understudied area of how systems change affects community demand and vice versa.^28^

This study provides rich qualitative insights into community dynamics around 2YL assimilation 4 years after MCV2 introduction when community consensus was still forming. The persistence of a coverage gap between MCV1 and MCV2 in Ghana suggests the demand and access barriers identified in this study remain relevant today. Indeed, the gap has been widening since 2018 as infant coverage has continued to improve while 2YL coverage has plateaued at 83%, aside from a general drop in coverage during the first year of the COVID-19 pandemic.^5^

Lessons from this analysis may also apply to vaccination platforms beyond 2YL, including perennially disappointing uptake for the adult influenza vaccine and COVID-19 vaccine. Broad disruption to childhood vaccination during the COVID-19 pandemic emphasizes the need for robust 2YL platforms to respond to the volume of catch-up vaccinations now required in many countries around the world. Since those disruptions stretched over multiple years, the 2YL platform may need to extend into the third and fourth years of life to cover all the children who missed routine doses over the course of the pandemic. Such programmatic challenges offer an opportunity to extend childhood vaccination from infancy through the age of formal enrollment in school or preschool, informed by the lessons of Ghana’s 2YL experiences, as reflected in the current analysis.

### Limitations

This study has several limitations. Because it was designed to complement the data from independent household and health facility baseline surveys, the caregiver FGDs relied exclusively on perceptions of caregivers and deliberately did not include perspectives of health care providers or other community stakeholders. Our results did confirm the findings of Nyaku et al. on broad awareness but limited specific knowledge about vaccine schedules among the general public.^6^ Secondly, the Health Belief Model, although appropriate for the objectives of a cross-sectional rapid assessment, missed the temporal and social dynamics of 2YL assimilation, for which later models of innovation and diffusion, like NPT, might have provided a better fit. Although we have done our best to frame our conclusions in this light, applying theoretical frameworks after the fact will always leave gaps in the analysis compared to research results if appropriate frameworks had been built into the initial stages of the study design. Future studies in vaccine introduction or life course expansion might consider longitudinal designs to track parallel assimilation processes among caregivers and care providers over time. Thirdly, even though 2YL vaccination was deliberately integrated into the 15-month weighing to boost demand and lower net costs to caregivers, our discussions focused exclusively on demand for 2YL vaccination without exploring the independent role of other services offered at the same visit. Finally, our analysis did not always find strong contrasts between designated comparison categories, such as gender or school enrollment status. Although this may point to true consensus across groups, it could also reflect a natural tendency of groups to gravitate toward dominant community narratives that may obscure meaningful differences in lived experience between groups.

## CONCLUSION

In conclusion, closing the 2YL vaccination coverage gap will ultimately require modifying embedded norms among caregivers and health care providers alike. Time is necessary but not sufficient to reach this goal. It requires the collective “work” of implementation together with community engagement to embed demand for expanded services while minimizing impediments to access. Such observations are consistent with systematic reviews of strategies to boost immunization demand across the lifecourse^29^ and are particularly relevant for the expansion of vaccination services in the second year of life and beyond.

## Notes

**Peer Reviewed**

**First published online:** June 20, 2023.

## References

[1] World Health Organization (WHO). *Global Vaccine Action Plan 2011–2020*. WHO;2013. Accessed May 1, 2023. http://www.who.int/immunization/global_vaccine_action_plan/GVAP_doc_2011_2020/en/

[2] Immunization Agenda 2030 (IA2030). *Immunization Agenda 2030: A Global Strategy to Leave No One Behind*. IA2030; 2021. Accessed May 1, 2023. http://www.immunizationagenda2030.org/

[3] World Health Organization (WHO). *Establishing and Strengthening Immunization in the Second Year of Life: Practices for Vaccination Beyond Infancy*. WHO; 2018. Accessed May 1, 2023. https://apps.who.int/iris/bitstream/handle/10665/260556/9789241513678-eng.pdf

[4] Introduction of measles-containing vaccine 2nd dose. World Health Organization. Accessed May 1, 2023. https://immunizationdata.who.int/pages/vaccine-intro-by-antigen/mcv2.html?ISO_3_CODE=&YEAR=

[5] WHO/UNICEF estimates of national immunization coverage. World Health Organization. Accessed May 1, 2023. https://www.who.int/teams/immunization-vaccines-and-biologicals/immunization-analysis-and-insights/global-monitoring/immunization-coverage/who-unicef-estimates-of-national-immunization-coverage 10.2471/BLT.08.053819 PMC2704038 19649368

[6] Nyaku M Wardle M Eng JV et al. Immunization delivery in the second year of life in Ghana: the need for a multi-faceted approach. Pan Afr Med J. 2017;27(Suppl 3):4. 10.11604/pamj.supp.2017.27.3.12182. 29296139 PMC5745947

[7] Tchoualeu DD Harvey B Nyaku M et al. Evaluation of the impact of immunization second year of life training interventions on health care workers in Ghana. Glob Health Sci Pract. 2021;9(3):498–507. 10.9745/GHSP-D-21-00091. 34593577 PMC8514031

[8] Rosenstock IM. Historical origins of the health belief model. Health Educ Monogr. 1974;2(4):328–335. 10.1177/109019817400200403 299611

[9] Wallace AS Ryman TK Dietz V. Overview of global, regional, and national routine vaccination coverage trends and growth patterns from 1980 to 2009: implications for vaccine-preventable disease eradication and elimination initiatives. J Infect Dis. 2014;210 (Suppl 1):S514–S522. 10.1093/infdis/jiu108. 25316875 PMC4663673

[10] Shuval HI Tilden RL Perry BH Grosse RN. Effect of investments in water supply and sanitation on health status: a threshold-saturation theory. Bull World Health Organ. 1981;59(2):243–248. 6972817 PMC2396042

[11] Peck M Gacic-Dobo M Diallo MS Nedelec Y Sodha SS Wallace AS. Global routine vaccination coverage, 2018. MMWR Morb Mortal Wkly Rep. 2019;68(42):937–942. 10.15585/mmwr.mm6842a1. 31647786 PMC6812836

[12] World Health Organization. Malaria vaccine: WHO position paper–March 2022. Weekly Epidemiological Record. 2022;97(09):60–78. Accessed May 1, 2023. https://www.who.int/publications-detail-redirect/who-wer9709-61%E2%80%9380

[13] World Health Organization (WHO). *Full Evidence Report on the RTS,S/AS01 Malaria Vaccine*. WHO; 2021. Accessed May 1, 2023. https://cdn.who.int/media/docs/default-source/immunization/mvip/full-evidence-report-on-the-rtss-as01-malaria-vaccine-for-sage-mpag-(sept2021).pdf?sfvrsn=c9737be_5

[14] Yeboah D Owusu-Marfo J Agyeman YN. Predictors of malaria vaccine uptake among children 6–24 months in the Kassena Nankana Municipality in the Upper East Region of Ghana. Malar J. 2022;21(1):339. 10.1186/s12936-022-04378-1. 36384655 PMC9666942

[15] Rogers EM. *Diffusion of Innovations*. Simon and Schuster; 2010.

[16] May C Finch T. Implementing, embedding, and integrating practices: an outline of normalization process theory. Sociology. 2009;43(3):535–554. 10.1177/0038038509103208

[17] Damschroder LJ Aron DC Keith RE Kirsh SR Alexander JA Lowery JC. Fostering implementation of health services research findings into practice: a consolidated framework for advancing implementation science. Implement Sci. 2009;4(1):50. 10.1186/1748-5908-4-50. 19664226 PMC2736161

[18] Atkins L Francis J Islam R et al. A guide to using the Theoretical Domains Framework of behaviour change to investigate implementation problems. Implement Sci. 2017;12(1):77. 10.1186/s13012-017-0605-9. 28637486 PMC5480145

[19] Birken SA Powell BJ Presseau J et al. Combined use of the Consolidated Framework for Implementation Research (CFIR) and the Theoretical Domains Framework (TDF): a systematic review. Implement Sci. 2017;12(1):2. 10.1186/s13012-016-0534-z. 28057049 PMC5217749

[20] Greenhalgh T Robert G MacFarlane F Bate P Kyriakidou O. Diffusion of innovations in service organizations: systematic review and recommendations. Milbank Q. 2004;82(4):581–629. 10.1111/j.0887-378X.2004.00325.x. 15595944 PMC2690184

[21] McEvoy R Ballini L Maltoni S O’Donnell CA Mair FS MacFarlane A. A qualitative systematic review of studies using the normalization process theory to research implementation processes. Implement Sci. 2014;9(1):2. 10.1186/1748-5908-9-2. 24383661 PMC3905960

[22] Bosu WK Ahelegbe D Edum-Fotwe E Kobina A Bainson Kobina Turkson P. Factors influencing attendance to immunization sessions for children in a rural district of Ghana. Acta Trop. 1997;68(3):259–267. 10.1016/S0001-706X(97)00094-6. 9492910

[23] Valente TW. Network Interventions. Science. 2012;337(6090):49–53. 10.1126/science.1217330. 22767921

[24] World Health Organization (WHO). *WHO Policy Statement: Multi-dose Vial Policy (MDVP). Revision 2014*. WHO; 2014. Accessed May 1, 2023. https://apps.who.int/iris/bitstream/handle/10665/135972/WHO_IVB_14.07_eng.pdf;sequence=1

[25] Chevalier R. Updating the behavior engineering model. Perform Improv. 2003;42(5):8–14. 10.1002/pfi.4930420504

[26] Bluestone J Ricca J Traicoff D Tchoualeu DD. It’s time to move beyond traditional health care worker training approaches. Glob Health Sci Pract. 2021;9(3):431–432. 10.9745/GHSP-D-21-00553. 34593570 PMC8514028

[27] Eccles M Grimshaw J Walker A Johnston M Pitts N. Changing the behavior of healthcare professionals: the use of theory in promoting the uptake of research findings. J Clin Epidemiol. 2005;58(2):107–112. 10.1016/j.jclinepi.2004.09.002. 15680740

[28] Nilsen P. Making sense of implementation theories, models, and frameworks. Implementation Sci. 2015;10:53. 10.1186/s13012-015-0242-0. 25895742 PMC4406164

[29] Brewer NT Chapman GB Rothman AJ Leask J Kempe A. Increasing vaccination: putting psychological science into action. Psychol Sci Public Interest. 2017;18(3):149–207. 10.1177/1529100618760521. 29611455

